# Dataset of the complete genome of *Streptomyces cavourensis* strain 2BA6PG^T^ isolated from sediment from the bottom of the salt lake Verkhnee Beloe (Buryatia, Russia)

**DOI:** 10.1016/j.dib.2022.108877

**Published:** 2023-01-02

**Authors:** Eric Tzyy Jiann Chong, De Chen Chiang, Keh Kheng Png, Elena Abidueva, Svetlana Zaitseva, Chenghang Sun, Ping-Chin Lee

**Affiliations:** a Biotechnology Research Institute, Universiti Malaysia Sabah, Jalan UMS, 88400 Kota Kinabalu, Sabah, Malaysia; b Faculty of Science and Natural Resources, Universiti Malaysia Sabah, Jalan UMS, 88400 Kota Kinabalu, Sabah, Malaysia; c Institute of General and Experimental Biology, Siberian Branch Russian Academy of Sciences, Ulan-Ude, Russia; d Institute of Medicinal Biotechnology, Chinese Academy of Medical Sciences and Peking Union Medical College, Beijing, China

**Keywords:** Complete genome, *Streptomyces cavourensis*, Sediment, Salt lake

## Abstract

The *Streptomyces cavourensis* strain 2BA6PG^T^ was isolated from sediment from the bottom of the salt lake Verkhnee Beloe (Buryatia, Russia). This strain's 7,651,223 bp complete genome has a high G + C content of 72.1% and consists of 7,069 coding sequences and 315 subsystems. The 16S ribosomal RNA of isolate 2BA6PG^T^ was most closely related to *Streptomyces cavourensis* strain NBRC 13026^T^ (98.91% identity), followed by *Streptomyces bacillaris* strain ATCC 15855^T^ (95.36%), *Streptomyces rhizosphaericola* strain 1AS2c^T^ (94.68%), and *Streptomyces pluricolorescens* strain JCM 4602^T^ (86.75%). These comparisons were supported by pairwise comparisons using average nucleotide identity (ANI) and DNA-DNA hybridization analysis. This is the first complete genome reported on *Streptomyces cavourensis* isolated from sediment from the bottom of the salt lake Verkhnee Beloe. The complete genome sequence has been deposited at the NCBI GenBank with an accession number CP101140.

**Specifications Table**

**Table:** 

Subject	Biology
Specific subject area	Microbiology, Genomics, Biotechnology
Type of data	Table, figures, and deposited data
How the data were acquired	The genome sequence was processed in Illumina HiSeq System and sequenced using a 2 × 150 bp paired-end sequencing kit
Data format	Raw, analyzed, and deposited
Description of data collection	*Streptomyces cavourensis* strain 2BA6PG^T^ was isolated from sediment from the bottom of the salt lake Verkhnee Beloe (Buryatia, Russia). Genomic DNA was isolated from a pure culture of *Streptomyces cavourensis* strain 2BA6PG^T^ and sequenced.
Data source location	*Streptomyces cavourensis* strain 2BA6PG^T^ was isolated from sediment from the bottom of the salt lake Verkhnee Beloe (Buryatia, Russia) (Latitude: 50.63 N, Longitude: 105.73 E).
Data accessibility	Data are deposited at the NCBI GenBank: https://www.ncbi.nlm.nih.gov/bioproject/849725 https://www.ncbi.nlm.nih.gov/biosample/29129586 https://www.ncbi.nlm.nih.gov/sra/PRJNA849725 https://www.ncbi.nlm.nih.gov/nuccore/CP101140

**Value of the Data**

• The complete genome of *S. cavourensis* strain 2BA6PG^T^ is useful for microbial taxonomy and ecological studies, especially in species distribution and identification.
• The complete genome of *S. cavourensis* strain 2BA6PG^T^ could be used for comparative genomic studies with other *Streptomyces* spp. for bioactive compound comparison.
• The complete genome of *S. cavourensis* strain 2BA6PG^T^ could provide essential data in discovering novel enzymes with desirable activities in industrial processes.

## 1 Objective

The *Streptomyces cavourensis* strain 2BA6PG^T^ was isolated from sediment from the bottom of the salt lake Verkhnee Beloe (Buryatia, Russia). An initial sanger sequencing using the 16S ribosomal RNA of this isolate showed that it was closely related to *Streptomyces cavourensis*. Since no complete genome of *Streptomyces cavourensis* has been reported from the salt lake Verkhnee Beloe, this strain's complete genome is sequenced for annotation and pairwise comparison.

## 2 Data Description

**Fig. 1:**
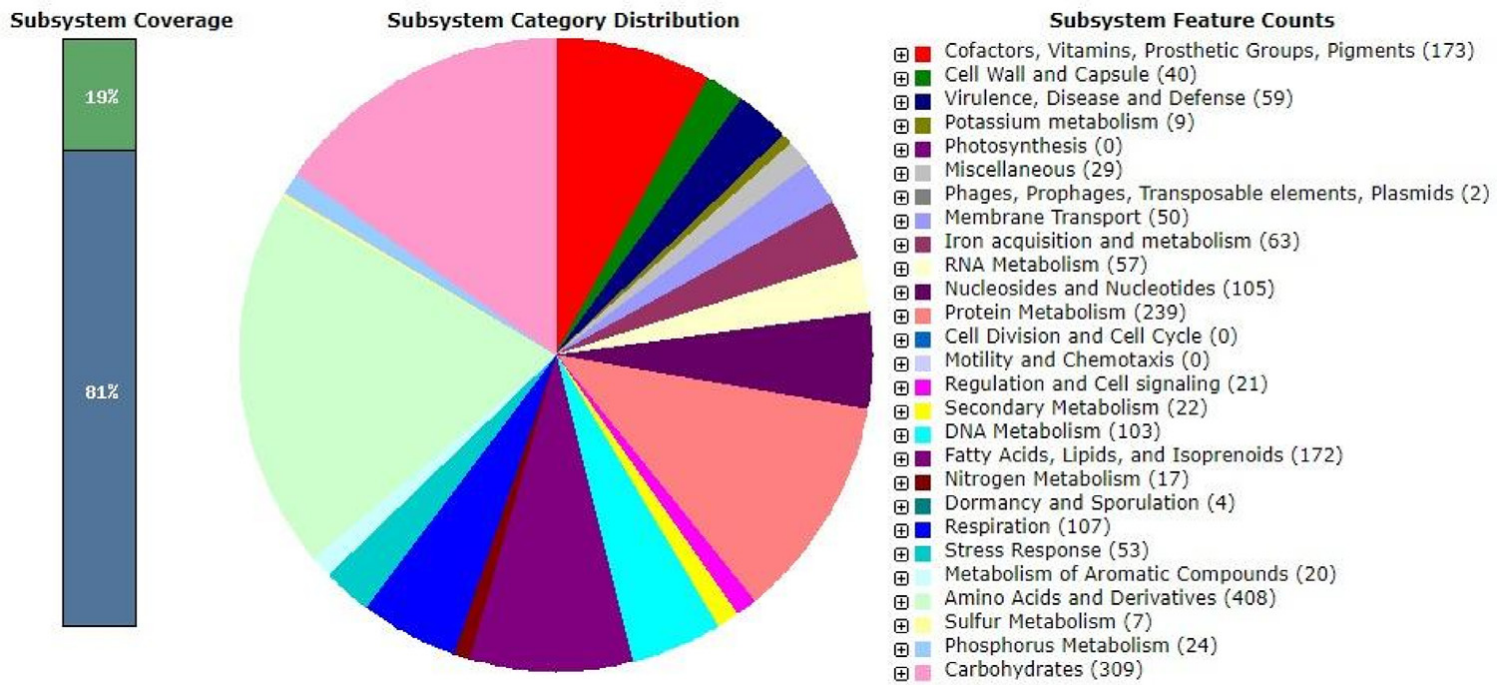
Subsystem feature counts of *S. cavourensis* strain 2BA6PG^T^ generated using RAST.

Natural products from plants and microbes have long been discovered as valuable sources of insights for new drug designs, especially the ones with precise efficacy in the medical treatment of infectious diseases [1], [2], [3]. Therefore, mining novel sources like the sediment of salt lakes, with vast and unique microbial biodiversity, will pave the way for discovering biological and chemical novelties. The *Streptomyces cavourensis* strain 2BA6PG^T^ was isolated from sediment from the bottom of the salt lake Verkhnee Beloe (Buryatia, Russia) (Latitude: 50.63 N, Longitude: 105.73 E). The complete genome of *Streptomyces cavourensis* strain 2BA6PG^T^ with a genome coverage of 20x was annotated using Rapid Annotations using Subsystems Technology (RAST). Its genome features are shown in Table 1 and Fig. 1. Overall, 7,651,223 bp of total bases were assembled with a G + C content of 72.1%. The genome is predicted with 315 subsystems and 7,069 coding sequences. The predicted number of RNAs was 87, with 40 tRNAs and 18 rRNAs. The highest subsystem feature count is related to amino acids and derivatives (*N* = 408).

**Table 1:** Genome features of *S. cavourensis* strain 2BA6PG^T^.

Attribute	*S. cavourensis* strain 2BA6PG^T^ value
Genome size (bp)	7,651,223
G + C content (%)	72.1
Number of subsystems	315
Number of coding sequences	7,069
Number of RNAs	87
tRNA	40
rRNA	18
others	29

Genome annotation was performed using antiSMASH version 6.1.1 to identify biosynthetic gene clusters (BGCs) of *S. cavourensis* strain 2BA6PG^T^. A total of 27 BGCs were identified, 13 of which matched known clusters with 70–100% similarity of isorenieratene, alkylresorcinol, melanin, valinomycin/montanastatin, SGR PTMs, bafilomycin B1, SAL-2242, keywimysin, desferrioxamine B, ectoine, coelichelin, streptobactin, and geosmin. The remaining 14 BGCs with less than 70% similarity were predicted to encode polyketide synthase (PKS) types I and II (*N* = 4), non-ribosomal peptide synthetase (NRPS) (*N* = 2), PKS + NPRS hybrid (*N* = 1), terpene (*N* = 1), ribosomally synthesized and post-translationally modified peptide (*N* = 1), terpene + saccharide hybrid (*N* = 1), PKS + saccharide hybrid (*N* = 2), PKS + NPRS + saccharide hybrid (*N* = 1), and other (*N* = 1). The potential and applications of these BGCs could be further explored.

**Fig. 2:**
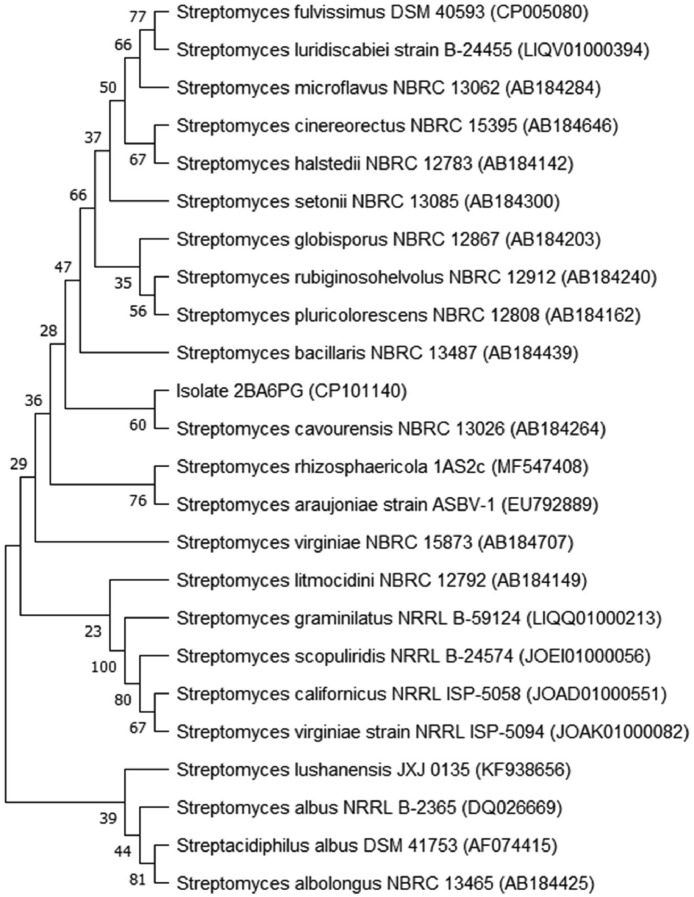
A phylogenetic tree constructed based on 16S rRNA sequences using the Neighbour-joining method in MEGA11. All sequences in the tree are derived from type strains. The number of nodes indicates the percentage support of 1000 bootstrap replicates.

The complete genome of *S. cavourensis* strain 2BA6PG^T^ did not contain the genome of other prokaryotes, as revealed by the ContEst16S tool integrated into EZBioCloud. A phylogenetic tree constructed using MEGA11 software based on the 16S ribosomal RNA (rRNA) showed that the isolate 2BA6PG^T^ was most closely related to *Streptomyces cavourensis* strain NBRC 13026^T^ (accession ID: AB184264) (Fig. 2).

Consistent with the phylogenetic tree, the average nucleotide identity calculation based on BLAST+ (ANIb) showed that the genome of isolate 2PA6PG^T^ had the highest similarity with *Streptomyces cavourensis* strain 1AS2a^T^ (98.91%), followed by *Streptomyces bacillaris* strain ATCC 15855^T^ (95.36%), *Streptomyces rhizosphaericola* strain 1AS2c^T^ (94.68%), and *Streptomyces pluricolorescens* strain JCM 4602^T^ (86.75%) (Table 2). This is further supported by the DNA-DNA hybridization (DDH) analysis, where the genome of isolate 2PA6PG^T^ had the highest DDH estimation (89.80%) and the lowest difference in G+C content (0.00%) with *Streptomyces cavourensis* strain 1AS2a^T^. It had the lowest DDH estimation (33.10%) and the highest difference in G+C content (0.44%) with *Streptomyces pluricolorescens* strain JCM 4602^T^. It is recommended that similairity values higher than 95–96% in ANIb analysis [4] and more than 70% in DDH estimation [5] are sufficient for species identification.

**Table 2:** DNA-DNA hybridization and ANIb studies of the isolate 2BA6PG^T^'s closely related species' genomes.

Species	ANIb*	DNA-DNA hybridization	
		DDH estimation	Difference in G + C content
*Streptomyces cavourensis* 1AS2a^T^ (CP024957)	98.91%	89.80%	0.00%
*Streptomyces bacillaris* ATCC 15855^T^ (CP029378)	95.36%	64.10%	0.18%
*Streptomyces rhizosphaericola* 1AS2c^T^ (SRZK01000100)	94.68%	57.50%	0.29%
*Streptomyces pluricolorescens* JCM 4602^T^ (BMUW01000001)	86.75%	33.10%	0.44%

*Average nucleotide identity calculation based on BLAST+.

## 3 Experimental Design, Materials, and, Methods

*S. cavourensis* strain 2BA6PG^T^ was isolated from the sediment sample. The samples were collected from the littoral zone of the salt lake Verkhnee Beloe (Buryatia, Russia) (Latitude: 50.63 N, Longitude: 105.73 E) during summer. Silt samples (0–0.5 cm) were collected with a sterile spatula into sterile containers and immediately placed into a refrigerator at 5–6 °C.

A 10-fold dilution was carried out after diluting the sample by 1 g in 10 mL of sterile distilled water. A 0.1% (v/v) solution of suppressing agents, 25 mg/mL nalidixic acid, 40 mg/mL cycloheximide, and 50 mg/mL potassium dichromate, was added. After that, 200 uL of the mixture was plated on Gause's synthetic agar (Huankai Microbial Sci. & Tech. Co., Ltd, Guangdong, China) supplemented with 2% sodium chloride and incubated at 27 °C. The plate was observed every 3 days for a month, and colonies were subcultured on a new agar plate. The *S. cavourensis* strain 2BA6PG^T^ was subcultured by streaking on the agar to obtain a single pure colony [6]. It was grown overnight at 27 °C in a nutrient broth with 2% sodium chloride before being subjected to genomic DNA isolation using the Bacteria Genomic DNA Isolation Kit (Canvax Biotech, Córdoba, Spain) according to the manufacturer's protocol.

For library preparation, a total of 200 µg of the genomic DNA was randomly fragmented by a benchtop focused-ultrasonicator (Covaris, Woburn, USA) to an average size of 300–350 bp. The fragments were treated with End Prep Enzyme Mix (New England Biolabs, Ipswich, USA) for end repairing, 5′ phosphorylation, and 3′ adenylated to add adaptors to both ends. A size selection of adaptor-ligated amplicons was then performed using cleanup beads and eight cycles of PCR amplification using P5 and P7 primers. The libraries were loaded onto an Illumina HiSeq System, and the genome was sequenced using a 2 × 150 bp paired-end sequencing kit (Illumina, San Diego, USA). The sequenced genome data were analyzed and assembled using the Galaxy Platform [7]. The raw reads were trimmed using Trimmomatic Galaxy version 0.36.6 [8] and assembled using SPAdes Galaxy version 3.15.4+galaxy0 [9], combined into 1 contig. The combined contig was subjected to annotation using RAST version 2 [10,11]. The BGCs of *S. cavourensis* strain 2BA6PG^T^ were annotated using antiSMASH version 6.1.1 [12].

The ContEst16S tool that was integrated into EZBioCloud [13,14] was used to determine any contamination of other prokaryotic genomes and to allocate the 16S rRNA sequence of the *S. cavourensis* strain 2BA6PG^T^. The 16S rRNA sequence (1528 bp) of the *S. cavourensis* strain 2BA6PG^T^ was used to construct a phylogenetic tree with closely related species of the genus *Streptomyces* as previously reported by Hoyos et al. [15] using a Neighbour-joining method with 1000 bootstraps in MEGA11 software [16]. Based on the phylogenetic tree, a pairwise comparison using the Average Nucleotide Identity (ANI) tool that was integrated into JSpeciesWS [17] and the DNA-DNA hybridization tool that was integrated into Genome-to-Genome Distance Calculator [18] were performed between the genome of isolate 2BA6PG^T^ and four other genomes of closely related species (*Streptomyces cavourensis* strain 1AS2a^T^ with an accession ID: CP024957, *Streptomyces bacillaris* strain ATCC 15855^T^ with an accession ID: CP029378, *Streptomyces rhizosphaericola* strain 1AS2c^T^ with an accession ID: SRZK01000100, and *Streptomyces pluricolorescens* JCM 4602^T^ with an accession ID: BMUW01000001).

## Ethics Statement

Not applicable.

## CRediT authorship contribution statement

**Eric Tzyy Jiann Chong:** Conceptualization, Formal analysis, Writing – original draft, Writing – review & editing. **De Chen Chiang:** Methodology, Formal analysis. **Keh Kheng Png:** Methodology. **Elena Abidueva:** Conceptualization, Resources, Writing – review & editing. **Svetlana Zaitseva:** Conceptualization, Resources, Writing – review & editing. **Chenghang Sun:** Conceptualization, Resources, Writing – review & editing. **Ping-Chin Lee:** Conceptualization, Resources, Supervision, Writing – review & editing.

## Declaration of Competing Interest

The authors declare that they have no known competing financial interests or personal relationships that could have appeared to influence the work reported in this paper.

## Data Availability

Dataset of complete genome of Streptomyces cavourensis strain 2BA6PG (Original data) (NCBI).
